# Studying the efficacy of low-dose colchicine on clinical outcomes of patients with STEMI: a randomized controlled trial

**DOI:** 10.1186/s43044-024-00515-0

**Published:** 2024-07-05

**Authors:** Mojtaba Yousefzadeh, Ali Khosrobeigi, Ayoub Salehi

**Affiliations:** Cardiology Research Department, Qom University of Medical Sciences, Qom, Iran

**Keywords:** Colchicine, STEMI, Inflammation

## Abstract

**Background:**

Numerous studies have underscored the essential role of inflammation across all stages of atherosclerosis. While various anti-inflammatory interventions have been implemented to mitigate inflammation-induced injuries, outcomes have been conflicting. Given the essential role of inflammation in these patients and limited data regarding the efficacy of low-dose Colchicine as an anti-inflammatory drug, we aimed to study the efficacy of low-dose Colchicine on clinical outcomes of patients with STEMI in Iran.

**Results:**

Participants presented with STEMI and qualified revascularization at Shahid Beheshti Hospital in Qom during 2022 and 2023 were included into the study. This study included 172 STEMI patients (114 males and 58 females) within the mean age of 58.93 ± 7.79. Results indicate that colchicine (2 mg for loading dose and 0.5 mg daily for 30 days) and placebo group were not significant differences in identical profiles regarding age and gender. Analyses revealed no significant differences in clinical outcome after the 40-day follow-up period.

**Conclusions:**

This study revealed that the addition of colchicine did not yield a significant benefit in enhancing the outcomes of patients with STEMI.

*Clinical trial registration*: This study was prospectively registered on Iranian registry of clinical trials, with registration number (IRCT20231001059578N1).

## Background

Ischemic heart diseases represent a global health threat, imposing substantial psychological and economic burdens on individuals [1]. Despite significant advancements in patient treatment and the widespread adoption of revascularization strategies, mortality and morbidity remain considerable [2]. Consequently, safeguarding myocardial health becomes pivotal in enhancing the prognosis of these patients by minimizing infarct size [3].

Numerous studies have underscored the essential role of inflammation across all stages of atherosclerosis. Inflammatory cells, notably neutrophils, followed by monocytes and macrophages, are recruited to the injured myocardium due to the secretion of inflammatory cytokines, exacerbating myocardial damage [4]. While various anti-inflammatory interventions have been implemented to mitigate inflammation-induced injuries, outcomes have been conflicting. The Canakinumab Anti-inflammatory Thrombosis Outcomes Study (CANTUS) study revealed a 15% reduction in the risk of cardiovascular diseases with interleukin-1β inhibition via canakinumab; however, this was counteracted by an increased incidence of fatal infections, leading to the drug's disapproval for cardiovascular disease prevention [5]. Conversely, the Cardiovascular Inflammation Reduction Trial (CIRT) study found that methotrexate had no significant impact on cardiovascular disease outcomes [6].

Colchicine emerges as a potent and widely used anti-inflammatory drug, known for its affordability, familiarity, and oral administration convenience. Currently approved for treating gout, Mediterranean fever, and pericarditis [7], colchicine accumulates within white blood cells, diminishing their mobility, chemotaxis, and endothelium attachment, thereby reducing inflammatory effects, cytokine release, and myocardial damage [8].

As previous studies reported significantly lower myocardial infarction prevalence in colchicine-treated gout patients compared to those without colchicine use [9], subsequent investigations demonstrated reduced creatine kinase myocardial band (CK-MB) levels in ST Elevation Myocardial Infarction (STEMI) patients undergoing Primary Percutaneous Coronary Intervention (PPCI), compared to a placebo group [10]. The COLCOT trial also affirmed that supplementing standard treatment with colchicine significantly lowered treatment costs for these patients [11].

Given the burden of STEMI in patients and limited data regarding the efficacy of low-dose Colchicine in Iran, we aimed to study the efficacy of low-dose Colchicine on clinical outcomes of patients with STEMI in Iran.

## Methods

A total of 172 patients enrolled in this double-blind randomized controlled clinical trial, with 86 participants in each group.

Informed consent from all participants and received backing from the Research and Technology Vice-Chancellor of Qom University of Medical Sciences, with approval from the university's Research Council and Ethics Committee.

Participants, aged between 18 and 80 years, presented with STEMI and qualified for PPCI or fibrinolytic revascularization within 12 h of pain onset at Shahid Beheshti Hospital in Qom during 2022 and 2023 were included into the study. Exclusion criteria encompassed unstable vital signs, documented colchicine sensitivity, severe liver or kidney disease, or chronic colchicine treatment.

Eligible patients were randomly assigned to either the colchicine or placebo group, employing a 1:1 ratio through stratified block randomization. An oral loading dose equivalent to 2 mg (two colchicine tablets or placebos) was administered, followed by 0.5 mg orally once daily for 30 days. The loading dose occurred pre-PCI and, if unfeasible, immediately post-PCI.

Our initial reperfusion strategy was the PPCI method for all patients, but due to the existing conditions or the crowding of the angiography department, if anticipated first medical contact (FMC)-to-device time more than 120 min, in less than 30 min from FMC, fibrinolytic was prescribed for them.

For patients who were transferred to the angiography department for the PPCI, after loading antiplatelets drugs, diagnostic angiography was performed first, and after wiring, a decision was made regarding the angioplasty method. Our priority was direct stenting, but depending on the conditions, other methods were also used.

The study's outcome measures included the severity of clinical symptoms, encompassing functional capacity and re-hospitalization necessity. Additionally, echocardiographic data, specifically left ventricular ejection fraction (LVEF) and left ventricular thrombosis, were assessed at the study's 40-day conclusion.

Transthoracic echocardiography (TTE) was conducted initially and after 40 days. TTE was performed with Siemens Acuson SC2000. Left ventricular end-diastolic diameter was measured in parasternal long-axis view, and left ventricular ejection fraction (LVEF) was calculated using modified biplane Simpson method of disks. In all prepared standard views (including parasternal long axis, parasternal short axis, apical 4 chamber, apical 5 chamber and apical 3 chamber), patients were evaluated for the presence of thrombosis. The initial TTE and also at the end of the study were performed by a same device and same operator.

Data were analyzed using SPSS 22 software. An independent *t* test was used for comparing continuous and Chi-square test for categorical variables. A *p* value < 0.05 was considered as statistically significant.

## Results

This study included 172 STEMI patients eligible for emergency revascularization, including 114 males and 58 females within the mean age of 58.93 ± 7.79. There were a total of 70 culprit lesions in LAD and 71 in RCA and 31 in LCX coronary arteries. Results indicate that both groups exhibited nearly identical profiles regarding age, gender distribution, revascularization procedure type, and the coronary vessel responsible for MI, indicating no significant differences between the groups (Table 1).

**Table 1:** Baseline characteristics of patients included into the study

Characteristic		Colchicine (*N* = 86)	Placebo (*N* = 86)	*P*-value
Age (Years)	Mean ± std	59.26 ± 7.92	58.60 ± 7.70	0.579
Gender	Male	55 (64%)	59 (68.6%)	0.519
	Female	31 (36%)	27 (31.4%)	
Culprit lesion	LAD	34 (39.5%)	36 (41.9%)	0.705
	LCX	14 (16.3%)	17 (19.8%)	
	RCA	38 (44.2%)	33 (38.4%)	
Revascularization type	PPCI	62 (72.1%)	59 (68.6%)	0.616
	Fibrinolytic	24 (27.9%)	27 (31.4%)	
Door to balloon time (minutes)	Mean ± std	68.14 ± 21.17	66.86 ± 22.49	0.748
Kllip class	I	59 (68.6%)	61 (70.9%)	0.355
	II	18 (20.9%)	21 (24.4%)	
	III	9 (10.5%)	4 (4.7%)	
	IV	0 (0%)	0 (0%)	
WBC	Mean ± std	9.08 ± 3.04	8.32 ± 3.65	0.139
Hb	Mean ± std	14.03 ± 2.55	14.72 ± 2.85	0.100

LAD: Left anterior descending artery, LCX: Left circumflex artery, RCA: Right coronary artery

Table 2 presents a comprehensive overview of the clinical outcomes for patients in the intervention and placebo groups. Analyses revealed no significant differences in readmission rates (2.3% in colchicine group and 2.3% in placebo group, *p*-value = 0.513), functional capacity (*p*-value = 0.857), left ventricular end-diastolic size (LVEDD) (*p*-value = 0.194), LVEF (*p*-value = 0.354), C-reactive protein (CRP) levels (*p*-value = 0.444), incidence of atrial fibrillation (AF) (*p*-value = 0.312), and left ventricular thrombosis after the 40-day follow-up period (*p*-value = 0.650).

**Table 2:** Clinical outcomes and adverse events in patients after 40 days post-intervention

Characteristic		Colchicine (*N* = 86)	Placebo (*N* = 86)	*p*-value
Readmission	No	84 (97.7%)	84 (97.7%)	0.513
	Once	1 (1.2%)	2 (2.3%)	
	Twice	1 (1.2%)	0 (0%)	
AF	No	85 (98.8%)	83 (96.5%)	0.312
	Yes	1 (1.2%)	3 (3.5%)	
LV thrombosis	No	83 (96.5%)	84 (97.7%)	0.650
	Yes	3 (3.5%)	2 (2.3%)	
Functional capacity	I	66 (76.7%)	69 (80.2%)	0.857
	II	14 (16.3%)	11 (12.8%)	
	III	3 (3.5%)	4 (4.7%)	
	IV	3 (3.5%)	2 (2.3%)	
Adverse effect	None	74 (86%)	77 (89.5%)	0.335
	Diarrhea	6 (7%)	2 (2.3%)	
	Nausea and vomiting	5 (5.8%)	7 (8.1%)	
	Infection	1 (1.2%)	0 (0%)	
Primary LVEF		37.15 ± 9.22	38.08 ± 8.75	0.498
LVEF after 40d		43.02 ± 6.33	43.95 ± 6.77	0.354
Primary CRP		26.55 ± 12.72	24.41 ± 11.12	0.242
CRP after 40d		6.38 ± 6.06	7.13 ± 6.84	0.444
LVEDD after 40d		50.93 ± 4.69	51.81 ± 4.17	0.194
In-hospital short-term outcome	Length of CCU stay	1.93 ± 1.18	2.13 ± 1.49	0.311
	In-hospital mortality	0 (0%)	1 (1.2%)	0.316
	Stroke	0 (0%)	1 (1.2%)	0.316
	Major bleeding	1 (1.2%)	0 (0%)	0.316
	re-MI	1 (1.2%)	2 (2.3%)	0.560
	Composite of In-hospital short-term outcome	2 (2.3%)	4 (4.7%)	0.406

Additionally, the study observed that complications resulting from colchicine were not statistically significant among the treated patients. Notably, only one patient experienced pneumonia infection, which did not necessitate hospitalization and was successfully managed through outpatient treatment.

## Discussion

The results of this study showed that the administration of Colchicine, with an initial dosage of 2 mg followed by 0.5 mg daily for 40 days, did not showed significant improvements in the treatment outcomes for patients with STEMI.

Colchicine is an anti-inflammatory drug whose effects and side effects are well known. This drug is known as an inexpensive drug that is easily available. It has been stated in various studies that this drug exerts its anti-inflammatory effects through various pathways, including: Destruction of microtubules and inhibiting the migration and movement of phagocytes [12], it also reduces the expression of E-Selectin in endothelial cells [13] and decreases the expression of L-Selectin in neutrophils, which prevents their invasion and attachment to the site of inflammation, and by interfering with the formation of NLRP3 inflammasome inhibits the release of IL-1β [14, 15].

Nevertheless, a noteworthy observation emerged regarding AF incidence. In the colchicine-treated group, only one patient experienced AF, who had a pre-existing AF rhythm during the initial emergency room visit. In contrast, the placebo group witnessed three new cases of AF rhythm post-MI. Upon re-analysis, excluding the aforementioned patient, a reduction in the p value to 0.08 was observed, hinting at a potential role for colchicine in preventing AF following myocardial infarction, aligning with prior studies emphasizing colchicine's role in AF prevention during heart surgeries [16].

Considering the need for anticoagulants in patients with AF rhythm, if administration of colchicine reduces the incidence of AF, this issue will be very important because STEMI patients are at a high risk of bleeding due to the need to take antiplatelet.

Another study highlighted colchicine’s capacity to significantly reduce MMP-9, NOX2, and TGF-β1 factors, pivotal in cardiac remodeling [17]. While our study could not measure these factors due to limitations, the assessment of left ventricular end-diastolic diameter at the 40-day mark revealed no distinction between the two groups.

Contrary to a recent study showing increased thrombosis incidence in a colchicine group, attributed to larger infarct size [18], our study’s conclusion indicated no variance in thrombosis occurrence between the intervention and placebo groups. Although our study did not investigate infarct size directly, the comparable EF and the involved vessel in both groups likely mitigated the impact of colchicine on left ventricular thrombosis.

Consistent with the COLIN trial and LoDoCo-MI results [19, 20], our study found no significant difference in CRP reduction between the colchicine and placebo groups. Conversely, despite a study on rats suggesting that short-term colchicine post-MI prevented left ventricular dilatation and improved LVEF [21], our study did not validate these findings.

Importantly, the use of colchicine demonstrated a lack of serious side effects in our study, and any observed side effects were mild and well-tolerated by the patients.

Several limitations warrant consideration in interpreting our results. These include a small sample size, the omission of cardiac magnetic resonance imaging (CMR) due to cost constraints, and the relatively short study duration. Future studies should address these limitations to enhance the generalizability and robustness of findings.

## Conclusions

This study revealed that the addition of colchicine did not yield a significant benefit in enhancing the outcomes of patients with STEMI. It is crucial to emphasize that drawing conclusions about the potential advantages or disadvantages of a drug based on a study with a limited sample size and a brief follow-up period is not feasible.

## Data Availability

Data can be provided (anonymized) upon reasonable request sent to the corresponding author.

## Abbreviations

CANTUS: Canakinumab anti-inflammatory thrombosis outcomes study
CIRT: Cardiovascular inflammation reduction trial
CK-MB: Creatine kinase myocardial band
STEMI: ST elevation myocardial infarction
PPCI: Primary percutaneous coronary intervention
LVEF: Left ventricular ejection fraction
LAD: Left anterior descending artery
LCX: Left circumflex artery
RCA: Right coronary artery
LVEDD: Left ventricular end-diastolic size
CRP: C-reactive protein
AF: Atrial fibrillation
CMR: Cardiac magnetic resonance imaging
FMC: First medical contact
TTE: Transthoracic echocardiography

## References

[1] Virani SS, Alonso A, Benjamin EJ, Bittencourt MS, Callaway CW, Carson AP (2020). Heart disease and stroke statistics—2020 update: a report from the American heart association. Circulation.

[2] Hartikainen TS, Sörensen NA, Haller PM, Goßling A, Lehmacher J, Zeller T (2020). Clinical application of the 4th universal definition of myocardial infarction. Eur Heart J.

[3] Heusch G (2020). Myocardial ischaemia–reperfusion injury and cardioprotection in perspective. Nat Rev Cardiol.

[4] Bochaton T, Lassus J, Paccalet A, Derimay F, Rioufol G, Prieur C (2021). Association of myocardial hemorrhage and persistent microvascular obstruction with circulating inflammatory biomarkers in STEMI patients. PLoS ONE.

[5] Ridker PM, Everett BM, Thuren T, MacFadyen JG, Chang WH, Ballantyne C (2017). Antiinflammatory therapy with canakinumab for atherosclerotic disease. N Engl J Med.

[6] Ridker PM, Everett BM, Pradhan A, MacFadyen JG, Solomon DH, Zaharris E (2019). Low-dose methotrexate for the prevention of atherosclerotic events. N Engl J Med.

[7] Tardif J-C, Kouz S, Waters DD, Bertrand OF, Diaz R, Maggioni AP (2019). Efficacy and safety of low-dose colchicine after myocardial infarction. N Engl J Med.

[8] Opstal TS, Hoogeveen RM, Fiolet AT, Silvis MJ, The SH, Bax WA (2020). Colchicine attenuates inflammation beyond the inflammasome in chronic coronary artery disease: a LoDoCo2 proteomic substudy. Circulation.

[9] Crittenden DB, Lehmann RA, Schneck L, Keenan RT, Shah B, Greenberg JD (2012). Colchicine use is associated with decreased prevalence of myocardial infarction in patients with gout. J Rheumatol.

[10] Deftereos S, Giannopoulos G, Angelidis C, Alexopoulos N, Filippatos G, Papoutsidakis N (2015). Anti-inflammatory treatment with colchicine in acute myocardial infarction: a pilot study. Circulation.

[11] Samuel M, Tardif J-C, Khairy P, Roubille F, Waters DD, Grégoire JC (2021). Cost-effectiveness of low-dose colchicine after myocardial infarction in the colchicine cardiovascular outcomes trial (COLCOT). Eur Heart J Qual Care Clin Outcomes.

[12] Martinez GJ, Celermajer DS, Patel S (2018). The NLRP3 inflammasome and the emerging role of colchicine to inhibit atherosclerosis-associated inflammation. Atherosclerosis.

[13] Tardif JC, Kouz S, Waters DD, Bertrand OF, Diaz R, Maggioni AP, Pinto FJ, Ibrahim R, Gamra H, Kiwan GS, Berry C (2019). Efficacy and safety of low-dose colchicine after myocardial infarction. N Engl J Med.

[14] Nidorf SM, Fiolet AT, Mosterd A, Eikelboom JW, Schut A, Opstal TS, The SH, Xu XF, Ireland MA, Lenderink T, Latchem D (2020). Colchicine in patients with chronic coronary disease. N Engl J Med.

[15] Chlorogiannis DD, Pargaonkar S, Papanagiotou P, Bakogiannis NC, Bakoyiannis C, Kokkinidis DG (2024). Inflammation, anti-inflammatory agents, and the role of colchicine in carotid artery stenosis. Vasa.

[16] Lennerz C, Barman M, Tantawy M, Sopher M, Whittaker P (2017). Colchicine for primary prevention of atrial fibrillation after open-heart surgery: systematic review and meta-analysis. Int J Cardiol.

[17] Suryono S, Rohman MS, Widjajanto E, Prayitnaningsih S, Wihastuti TA, Oktaviono YH (2023). Effect of colchicine in reducing MMP-9, NOX2, and TGF-β1 after myocardial infarction. BMC Cardiovasc Disord.

[18] Mewton N, Roubille F, Bresson D, Prieur C, Bouleti C, Bochaton T (2021). Effect of colchicine on myocardial injury in acute myocardial infarction. Circulation.

[19] Akodad M, Lattuca B, Nagot N, Georgescu V, Buisson M, Cristol J-P (2017). COLIN trial: value of colchicine in the treatment of patients with acute myocardial infarction and inflammatory response. Arch Cardiovasc Dis.

[20] Hennessy T, Soh L, Bowman M, Kurup R, Schultz C, Patel S (2019). The low dose colchicine after myocardial infarction (LoDoCo-MI) study: a pilot randomized placebo controlled trial of colchicine following acute myocardial infarction. Am Heart J.

[21] Fujisue K, Sugamura K, Kurokawa H, Matsubara J, Ishii M, Izumiya Y (2017). Colchicine improves survival, left ventricular remodeling, and chronic cardiac function after acute myocardial infarction. Circ J.

